# The Poetry of Poverty

**Published:** 1876-06

**Authors:** 


### *THE POETRY OF POVERTY.*

HOOD'S "Song of the Shirt," sent a thrill through society, and no nobler inscription could have been placed over his grave than the record that he sang that song. He also did good, perhaps even greater service, by his "Bridge of Sighs," a poem which has set most men thinking, and which has, without doubt, prompted many to assist in reclaiming the lost, for who, without a thrill, can read those burning words, describing the end of "One More Unfortunate," and the outburst,

> Picture it, think of it,

> Dissolute man!

Or the pathos of the last stanza—

> Cross her hands humbly,
> As if praying dumbly,
> Over her breast—
> Owning her weakness,
> Her evil behavior,
> And leaving, with meekness,
> Her sins to her Saviour.

But Hood was not the first who claimed the right of brotherhood for the pauper. He was preceded by one from whom there is little doubt he caught the inspiration.

In 1841, two years before the "Song of the Shirt" made itself heard in the pages of "Punch," there appeared a little, unassuming volume of "Rhymes and Roundelays," by T. Noel, and published by William Smith, 113 Fleet Street, London. Who Mr. Noel was we have been unable to learn, but in answer to recent inquiries were told "he died at Brighton ten or twelve years ago." The poem attracted but little attention here; we first knew of its existence from an American collection, where it appeared with the name of Thomas Hood appended, and to whom it might, from its rhythm, be very naturally attributed. It is entitled

### THE PAUPER'S DRIVE.

> There's a grim one-horse hearse in a jolly round trot;
> To the churchyard a pauper is going, I wot;
> The road it is rough, and the hearse has no spring,
> And hark to the dirge that the sad driver sings:
> "Rattle his bones over the stones,
> He's only a pauper whom nobody owns!"

Oh, where are the mourners? alas! there are none;
He has not left a gap in the world, now he's gone;
Not a tear in the eye of child, woman, or man;
To the grave with his carcass as fast as you can;
"Rattle his bones over the stones,
He's only a pauper whom nobody owns!"

What a jolting and creaking, and splashing and din!
The whip, how it cracks! and the wheels how they spin!
How the dirt right and left o'er the hedges is hurl'd!
The pauper at length makes a noise in the world!
"Rattle his bones over the stones,
He's only a pauper whom nobody owns!"

Poor pauper defunct! he has made some approach
To gentility, now that he's stretched in a coach;
He's taking a drive in his carriage at last;
But it will not be long if he goes on so fast!
"Rattle his bones over the stones,
He's only a pauper whom nobody owns!"

You bumpkin! who stare at your brother convey'd,
Behold what respect to a cloddy is paid.
And be thankful to think, when by death you're laid low,
You've a chance to the grave like a gemmen to go,
"Rattle his bones over the stones,
He's only a pauper whom nobody owns!"

But a truce to this strain, for my soul it is sad,
To think that a heart in humanity clad
Should make, like the brutes, such a desolate end,
And depart from the light without leaving a friend!
Bear softly his bones over the stones,
Though a pauper, he's one whom his Master yet owns!

Neither was Hood the last to write on the subject; there have been many since his time who have sung the songs of poverty. Some have sung them well, most but indifferently, and but few that can be remembered. That which we consider the best appeared in a publication but little known to the general public—"Cope's Tobacco Plant," a monthly journal issued by a wholesale tobacconist at Liverpool. Its mission is to set forth praises of the weed; but the editor, Mr. Fraser, is a man of such marvellously good taste and knowledge, that we never take up "The Tobacco Plant" without finding much that is exceedingly interesting apart from the fumes of tobacco. A few months ago there appeared an exquisitely pathetic piece of real poetry, descriptive of a pauper's last night. And, although non-smokers can not realize the fact, there is the universal testimony of all who indulge in the use of the weed that it exercises a soothing, quieting influence, making the smoker for a time oblivious to his cares, and creating for him a joy known only to the initiated. We have no doubt, therefore, that the author, Mr. H. Lloyd, expresses no more than the truth when he says—

'Tis hard to spare a penny to find
The weed, with starvation before and behind,
And yet it is harder without it.

And perhaps the quantity of tears shed over the grave of a pauper were never more accurately gauged than when he tells us that they might all be "kept in a bottomless measure." We give the whole of the poem except the last verse, which, perhaps, the author may himself think it worth while to omit when he reprints.

### The Casual's Last Pipe.

"But little they know, and less they care,
These crowds that pass in the glitter and glare.
So proud of their high position,
What it is to be housed in the parish fold,
And fed on hunger and clothed with cold,
With the last crust gone, and the last rag sold,
And the soul on the edge of perdition.

"I could preach 'em a sermon, but need my breath
To blow my one comfort 'twixt this and death;
From a bowl that's black as my heart is;
Yes, yes, old pipe, thou art black and foul,
Just fit to be stuck in my ugly jowl;
Your one eye glows like the eye of an owl,
Unfit for respectable parties..

"Ah, no! ah no! 'twas not always so;
The heavens were bright as the earth below,
And life was all pleasant sailing;
With my own dear Emma, and babes as well,
This earth was heaven, and now it is hell;
For right from the top of my pride I fell
To a place of most doleful wailing.

"It was the bank, the bank that broke:
Thousands went down at that cursed stroke,
As I went down to my ruin.
The bland directors with simper and bow,
I cursed them then, and I curse them now;
Each heart and soul and placid brow
I curse, for it was their doing.

"They were sorry to say that things weren't right,
And so I am sitting here to-night;
Who wonders my feelings are hateful?
Emma died first, for the food she ought
To have had was lost in the prime old port
The directors swallow'd to help them discuss
The surest means to satisfy us,
For which we were very grateful.

'My children three, who were weak and small,
Held up for awhile, but death had them all;
They followed their mother to heaven.
Better, ah, better, yes, every way,
That they should lie under the foul black clay,
And soundly sleep till the judgment day,
Than strive as I have striven.

"Better be laid in a nameless grave
Than live to see their old father a knave—
A knave of the worst condition;
Which means a pauper, for knavery drest
In costly clothing is fit for the best,
Aye, fit to be the lauded guest
Of men in the highest position.

"'Tis hard to spare a penny to find,
The weed, with starvation before and behind,
And yet it is harder without it;
For nothing can give me comfort but this:
For a moment it bringeth forgetfulness.
'Tis the only thing on earth that I bless—
Put yourselves in my place, ye who doubt it.

"And as to starvation, well, I can say
I've lived upon that for many a day,
Till my skin is too big for its scaffold.
There'll be the less to bury, no doubt.
When they cover me in, and carry me out;
There isn't a worm, but'll turn up his snout,
At finding himself so baffled.

"And that's how I'm sitting here to-night,
A starving old wretch, with my pipe alight,
A warning to all generations;
Ready to cringe, or grovel, or thrust
My nose in the dust for a mouldy crust,
Or beg or steal, or borrow on trust
For poverty's rotten rations.

"The directors were shaken a bit, but somehow
They righted themselves and are pretty well now
That little affair is forgotten;
And the widow's wail and the orphans's cries
Are blown to the winds, and the sun in the skies
Looks down from above, and shows no surprise
That society's heart is so rotten.

"Gone out, my old pipe. Well, I need not wait,
For I hear them unbarring the dingy gate;
I wish 'twould close on me forever.
Lie safe in my bosom till morning comes,
To find you again in my toothless gums,
While I seek for refuse in filthy slums,
And warm my old bones with a shiver."

Now, what the pauper dreamed in his dreams
I can not tell, nor you; but it seems
That his sleep was sound and lasting.
When the summons came to go breaking stones.
They found just a bundle of senseless bones;
And the coroner said, in plainest tones,
"The cause of his death was fasting."

One hand was grasping his pipe full fast,
As fighting death for that till the last,
His cheapest and dearest treasure.
However, they bundled him into a shell,
And hurried him off to his grave pell-mell;
And all the tears that over him fell
Might be kept in a bottomless measure.

—*Whittaker's Journal.*

